# Visual over auditory superiority in sensorimotor timing under optimized condition

**DOI:** 10.3389/fpsyg.2022.1048943

**Published:** 2022-11-24

**Authors:** Liying Zhan, Yingyu Huang, Zhihan Guo, Junkai Yang, Li Gu, Shengqi Zhong, Xiang Wu

**Affiliations:** Department of Psychology, Sun Yat-sen University, Guangzhou, China

**Keywords:** audition, ecological, sensorimotor, timing, vision

## Abstract

Auditory over visual advantage in temporal processing is generally appreciated, such as the well-established auditory superiority in sensorimotor timing. To test for a possible visual superiority in temporal processing, here, we present a data set composed of a large 60 subjects sample and a data set including eight smaller samples of approximately 15 subjects, showing that synchronization to a temporally regular sequence was more stable for a visual bouncing ball (VB) than for auditory tones (ATs). The results demonstrate that vision can be superior over audition in sensorimotor timing under optimized conditions, challenging the generally believed auditory superiority in temporal processing. In contrast to the auditory-specific biological substrates of timing in sensorimotor interaction, the present finding points to tight visual-motor cortical coupling in sensorimotor timing.

## Introduction

Audition has long been considered to be specialized for temporal processing compared with the visual advantage in spatial processing (Repp and Penel, 2002; Guttman et al., 2005; Grahn et al., 2011; Chen and Vroomen, 2013; Comstock et al., 2021). This general auditory superiority in processing temporal information, however, was questioned by evidence found in recent sensorimotor timing research. In the classic sensorimotor timing task of moving in time with a perceived pulse, e.g., tapping finger in synchrony with isochronous sequences, it is well known that synchronization is much more stable for auditory tones (ATs) than for visual flashes (Repp, 2005; Patel, 2014). Synchronization stability measures the variability of tapping, with higher stability indicating that tapping is less variable. Inspired by the ecological relevance of moving visual stimuli as suggested by Repp and Penel: “A flashing light is not a common visual experience, whereas moving objects and organisms are ubiquitous” (Repp and Penel, 2004), recent research has employed periodically moving visual stimuli and showed improved visuomotor timing performance compared with conventional visual flashes (Hove et al., 2010, 2013a,2013b; Varlet et al., 2014; Iversen et al., 2015).

Among the efforts to improve visuomotor timing by employing moving visual stimuli, a study designed a bouncing ball that simulated the effect of gravity by using a uniformly varying velocity (Gan et al., 2015). This gravity bouncing ball yielded the first evidence that visuomotor synchronization was not less stable than audiomotor synchronization. Noticeably, the results of Gan et al. (2015) also revealed a weak effect that, except for the unnaturally fast condition [300 ms inter-onset interval (IOI)] in which the subjects reported that the fast ball sequence looked unnatural, synchronization to the gravity bouncing ball was consistently more stable than synchronization to the AT when the tempi were in the range appropriate for tapping (IOIs ranging from 500 to 900 ms). Although the weak visual over auditory effect observed in Gan et al. (2015) had a small effect size and did not always reach a statistically significance level of *p* < 0.05, it inspired the present work to test for the possibility of a visual superiority in sensorimotor timing, for both theoretical and statistical considerations.

Theoretically, the possibility of a visual superiority raises a key question regarding the understanding of biological substrates of sensorimotor timing: whether visuomotor timing could outperform audiomotor timing. For instance, could sensorimotor synchronization be more stable for the visual than for the auditory system. Vision has been suggested to be much less trustworthy in sensorimotor timing than audition (Repp and Penel, 2002; Grahn et al., 2011). Indeed, the auditory system has higher temporal resolution than the visual system (Holcombe, 2009). However, vision is the primary sense in humans and efficient visuomotor timing is vital for us to interact with environments. We have hypothesized that visuomotor timing may not be less trustworthy than audiomotor timing (Gan et al., 2015), based on two considerations. First, compared with processing time intervals in an absolute and unrelated manner (which is referred to as duration-based timing), temporal regularities enables time intervals to be organized and encoded relative to expected points in time and improve the processing of temporal information (which is referred to as beat-based timing) (Povel and Essens, 1985; Large and Jones, 1999; Patel et al., 2005; Grahn and Rowe, 2009; Teki et al., 2011). The organization of temporal patterns as reflected in beat-based timing may not necessarily be restricted by the lower temporal resolution of the visual system. Second, the visual information in our environments commonly contains spatiotemporal changes, whereas static visual flashes are the most often adopted visual stimuli in previous sensorimotor timing researches (Repp, 2005; Repp and Su, 2013). Therefore, in a beat synchronization task (Repp, 2005; Patel, 2014) employing moving visual stimuli with strong ecological relevance (Gan et al., 2015; Huang et al., 2018; Gu et al., 2019), visuomotor timing should be substantially enhanced. The behavioral superiority of audition to vision in sensorimotor timing has been an essential basis for the proposed tight connections between the auditory and motor cortices but not between the visual and motor cortices (Thaut et al., 1999; Zatorre et al., 2007; Patel, 2014). Evolutionary hypotheses of sensorimotor timing also consider an auditory-specific mechanism driven by vocal learning (Patel et al., 2009; Patel, 2014). Whereas the auditory-specific hypotheses emphasize tight auditory-motor cortical coupling for sensorimotor timing, the recent finding of improved visuomotor timing performance by moving visual stimuli has motivated a reconsideration of the auditory-specific hypothesis that tight connections might also exist between the visual and motor cortices (Repp and Su, 2013; Gan et al., 2015; Iversen and Balasubramaniam, 2016). In this regard, we suggest that the existing evidence of improved visuomotor timing performance does not necessarily require a renewal of the auditory-specific mechanisms, unless visuomotor timing can outperform audiomotor timing. Improved visuomotor timing performance, even as good as performance of audiomotor performance, may not necessarily involve tight visual-motor cortical coupling. A pioneer study of neural plasticity has shown that visual behavior can be mediated by the auditory pathway when the visual cortex is inactive (von Melchner et al., 2000). Therefore, it could be a more efficient manner for the brain to utilize the audiomotor pathway to process visual temporal information, instead of relying on the visuomotor pathway that has been supposed to be much less efficient than the audiomotor pathway. However, if visuomotor timing could outperform audiomotor timing, the visual superiority would be difficult to be fully accounted for by utilizing the audiomotor pathway and thus indicates the existence of tight visual-motor cortical coupling in sensorimotor timing.

Statistically, the data of Gan et al. (2015) have only provided a weak clue for, but did not demonstrate a visual superiority in sensorimotor timing. The sampling variability is commonly appreciated (Efron, 1979; Hunter and Schmidt, 1990), and recent concerns have emphasized the importance of replication in evaluation of an experimental effect, by using larger samples or examining effects across smaller samples (Cumming, 2014; Open Science Collaboration, 2015). In this regard, a sample of approximately 15 subjects that is typically adopted in sensorimotor timing studies including Gan et al. (2015) could have missed capturing the statistical significance of the visual over auditory effect, in terms of a statistical criterion of *p <* 0.05.

Therefore, we hypothesized that visuomotor timing would outperform audiomotor timing, which is a key to understanding biological substrates of sensorimotor timing: the visual over auditory superiority would point to tight visual-motor cortical coupling. We predicted that synchronization to the gravity bouncing ball would be more stable than synchronization to the AT. To this end, the present study examined the possible visual over auditory effect in two data sets, both of which used the same task of synchronizing finger taps to a temporally regular sequence and the same sequence types composed of ATs or visual gravity bouncing balls (Gan et al., 2015). The first data set were newly collected and included a large 60 subjects sample. We referred this data set as the large sample-size data set. The gravity bouncing ball designed in Gan et al. (2015) has been adopted in the following studies (Huang et al., 2018; Mu et al., 2018; Gu et al., 2019). The second data set was composed of the eight smaller samples that had been published in these studies (Gan et al., 2015; Huang et al., 2018; Mu et al., 2018; Gu et al., 2019). Each data sample had approximately 15 (13–17) subjects, and we referred the second data set as the small sample-size data set. Together, inspired by the weak evidence of a visual superiority in sensorimotor timing observed in our initial study (Gan et al., 2015), in the present work we attempted to look for strong evidence by using a larger sample and *via* cumulating evidence across smaller samples (Lipsey and Wilson, 2001; Cumming, 2013, 2014; Open Science Collaboration, 2015).

## Materials and methods

### Participants

The large sample-size data set with sixty subjects had 17 males (mean age ± SD 21.7 ± 1.8). Sixteen subjects reported musical experience over 5 years. (In the large sample-size data set, the data of 53 subjects were from a Gene-Brain-Behavior Project). The small sample-size data set is summarized in Table 1. All subjects were right-handed, had normal hearing and had normal or corrected-to-normal vision. The research protocols in this study followed the tenets of the Declaration of Helsinki and were approved by the Institutional Review Board of Psychology Department of Sun Yat-sen University. All subjects gave written informed consent.

**TABLE 1:** Subjects’ information of the small sample-size data sets.

Samples	Experiments	Number of subjects	Number of males	Mean age±SD (years)	Number of subjects with musical experience over 5 years	Published study
1∼2	1	15	4	22.7 ± 2.9	3	Gan et al., 2015
3∼5	1	14	4	22.6 ± 1.6	3	Gan et al., 2015
6	2	17	2	24.5 ± 2.2	1	Mu et al., 2018
7	3	15	1	21.8 ± 2.8	3	Huang et al., 2018
8	4	13	2	21.7 ± 3.5	0	Gu et al., 2019

The eight smaller data samples were from four experiments conducted in four published studies.

### Power analysis

The sample size of the large sample-size data set was estimated by *a priori* power analysis performed using G*Power 3 (Faul et al., 2007). The analysis was in accordance to the stability difference between the AT and the visual bouncing ball (VB) (i.e., the crucial effect investigated in the present study) reported in previous studies (Gan et al., 2015; Huang et al., 2018; Mu et al., 2018; Gu et al., 2019) (i.e., the published Samples 1–8). The effect computed by a meta-analysis was *g* = –0.39. Based on this effect size, the alpha level of *p* < 0.05 (two tailed), and the power of 0.80, 54 subjects were required.

Note that the current power analysis was not performed for the individual smaller samples in the small sample-size data set, because power analyses have been reported in the corresponding studies (See below description of original experimental purposes of individual data samples).

### Stimuli and procedure

**FIGURE 1:**
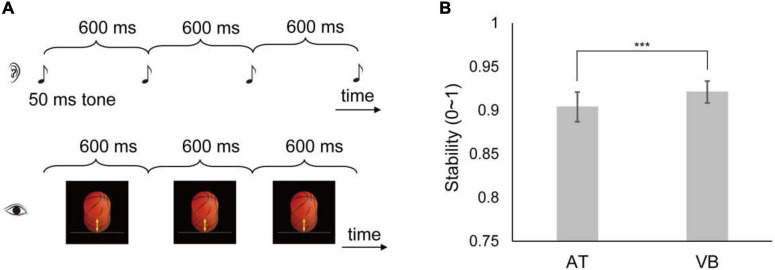
Illustration of the experimental stimuli and synchronization stability results in the large sample-size data set. **(A)** The subjects tapped along with an isochronous sequence composed of an auditory tone (AT) (up: the AT sequence) or a visual bouncing ball (VB) (below: the VB sequence). Three cycles of the 600 ms inter-onset interval (IOI) sequences are shown. [The images are adapted from Huang et al. (2018)]. **(B)** Shown are the synchronization stabilities of the AT and VB sequences. The error bar indicates ±95% CIs. ***Indicates *p* < 0.001.

In both data sets, the subjects were asked to tap in synchrony with a temporally regular (isochronous) sequence using the index finger of their preferred hand on a computer keyboard key. The sequence had 32 events in the large sample-size data set. In the small sample-size data set, the sequence had 30 events in Sample 6, 32 events in Sample 8, and 55 events in the other samples. The IOI was 600 ms in the large sample-size data set. In the small sample-size data set, the IOI was 500 ms for Sample 3, 700 ms for Samples 4 and 8, 900 ms for Sample 2, and 600 ms for the other samples. Each data set had two types of sequence, the AT sequence composed of a 50 ms 600 Hz tone and the VB sequence composed of a periodically bouncing ball (Figure 1A). The construction of the stimuli has been detailed in Gan et al. (2015), and we concisely described the bouncing ball here. In an IOI, a realistic orange basketball with 1.74 cm in diameter continually moved 0.92 cm (movement distance) down and then moved up to the initial position at the center of the computer screen. The bouncing ball had a uniformly varying velocity with the acceleration of 0.20 m/s^2^, thus simulating the effect of gravity. Each sequence types were repeated six times and the order of sequence types was counterbalanced across subjects.

Two notes about sequence types would be mentioned. First, all experiments were originally designed for specific experimental purposes and had more than two sequence types. Only the AT and VB sequences were adopted in the present study for the investigation of the visual over auditory superiority. Second, it is suggested that auditory sequences of IOIs ranging from 300 to 900 ms are appropriate to tap with (Repp, 2005; Hove et al., 2010; Repp and Su, 2013; Iversen et al., 2015). Gan et al. (2015) found that the VB sequence was appropriate to synchronize for IOIs ranging from 500 to 900 ms, except for the 300 ms IOI in which the movement was reported to be unnaturally fast. Therefore, the AT or VB sequences with IOIs from 500 to 900 ms were adopted in the current study and the IOIs were not distinguished when evaluating effects across data sets.

### Data analyses

The present study focused on the analysis of synchronization stability, because (1) it has been suggested that the stability is more sensitive in identifying individual differences of synchronization performances as compared with the mean asynchrony (Hove et al., 2013a; Dalla Bella and Sowiński, 2015), and (2) the improvement of visuomotor timing performance by periodically moving visual stimuli was primarily revealed by the stability (Hove et al., 2010, 2013a; Gan et al., 2015; Iversen et al., 2015; Huang et al., 2018; Mu et al., 2018; Gu et al., 2019). Synchronization stability was calculated for the large sample-size data set. For the small sample-size data sets that have been published, the values of synchronization stability have been reported and were adopted in the present study. Here we concisely describe the analysis method of sensorimotor synchronization data, which has been detailed in previous published studies (Gan et al., 2015; Huang et al., 2018; Mu et al., 2018; Gu et al., 2019). The raw tapping data consisted of sequences of tap times. The difference between the time of a tap and the time of the corresponding stimulus onset (asynchrony) was measured by the relative phase (RP) on a unit circle (–pi to pi. 0 indicated perfect alignment between taps and events; negative and positive values indicated taps preceding or following events, respectively; and ±pi indicated taps midway between events). Synchronization stability was indexed by R, which was the length of the resultant (i.e., average of vectors) of the RPs and was calculated by abs (sum (exp (i*RP))/*n*) (*n* indicated the number of the RPs). R ranged from 0 (unstable tapping with uniformly distributed RPs) to 1 (perfectly stable tapping with a unimodal distribution of RPs). Correspondingly, mean asynchrony was indexed by the angle of the resultant of the RPs and was calculated by angle (sum (exp (i*RP))/*n*).

## Results

We first examined synchronization stabilities of the AT sequence and the VB sequence in the large sample-size data set. The results are shown in Figure 1B. The stability was significantly lower for the AT sequence than for the VB sequence [mean difference = –0.02, 95% CI = (–0.03, –0.01), *t*_59_ = –2.74, *p* = 0.008, Cohen’s *d*_*z*_ = –0.35].

**FIGURE 2:**
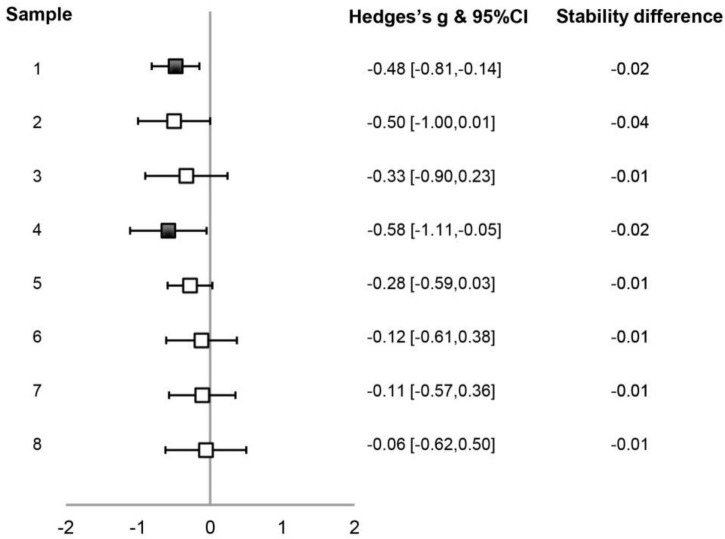
Illustration of the differences between synchronization stabilities of the auditory tone (AT) and the visual bouncing ball (VB) sequences in the small sample-size data set. For each data sample, Hedges’s *g* of the stability difference between the two sequence types and the 95% CIs are plotted (filled symbols indicating *p* < 0.05), with the values listed on the right of the plot. The value of the mean stability difference is also indicated. Other conventions are as in Figure 1 and Table 1.

We then examined synchronization stabilities of the AT and VB sequences in the small sample-size data set. The difference between synchronization stabilities of the AT and VB sequences is presented in Figure 2 for individual data samples. While the variability among samples can be seen and the difference only reached a statistical level of *p <* 0.05 in two data sets (1 and 4), it was also noticeable that the stability was lower for the AT than for the VB sequence in all samples. A random-effect meta-analysis using the Schmidt–Hunter method (Hunter and Schmidt, 1990; Viechtbauer, 2010) was further carried out to evaluate the effect across data samples. The effect size of the difference between synchronization stabilities of the AT and VB sequences was calculated as Hedge’s *g* for each data sample (Borenstein et al., 2011). The analysis revealed a reliable difference between the stabilities of the AT and VB sequences across samples [*g* = –0.32, 95% CI = (–0.48, –0.17), *z* = –4.06, *p* < 0.001], suggesting that synchronization was more stable for the VB than for the AT sequence. Note that Samples 1–5 were treated as separate samples in the analysis, which could have improperly estimated the precision of the overall effect (Borenstein et al., 2011) since Samples 1 and 2 were from a sample of same subjects and Samples 3–5 were also from a sample of same subjects. We therefore conducted a further analysis using composite effects by computing a combined effect across Samples 1 and 2 and a combined effect across Samples 3–5, which confirmed the reliability of the stability difference between the AT and VB sequences [*g* = –0.32, 95% CI = (–0.49, –0.15), *z* = –3.72, *p* < 0.001].

Moreover, we also examined mean asynchronies between the AT and VB sequences. For the large sample-size data set, mean asynchrony was more negative for the AT sequence than for the VB sequence, and the difference reached significance with the upper end of the CI being –0.0002 [mean difference = –0.17, 95% CI = (–0.33, –0.0002), *t*_59_ = –2.00, *p* = 0.050, Cohen’s *d*_*z*_ = –0.26]. For the small sample-size data set, a meta-analysis did not show a significant difference between the two sequence types [*g* = 0.22, 95% CI = (–0.04, 0.49), *z* = 1.68, *p* = 0.092]. For individual samples, mean asynchrony in the AT sequence was more negative than mean asynchrony in the VB sequence in three samples (2, 6, and 8), and was less negative in five samples (1, 3, 4, 5, and 7). The mean asynchrony difference in Sample 6 was significant [mean difference = –0.54, 95% CI = (–0.80, –0.28), *t*_59_ = –4.39, *p* < 0.001, Cohen’s *d*_*z*_ = –1.06]. The results of mean asynchrony did not show consistent differences across data sets, consistent with previous results that improvement of visuomotor timing was primarily revealed by the measure of stability (Hove et al., 2010, 2013a; Gan et al., 2015; Iversen et al., 2015; Huang et al., 2018; Mu et al., 2018; Gu et al., 2019).

## Discussion

By a data set with a large sample of 60 subjects and a data set with eight smaller samples of approximately 15 subjects, the present results established that synchronization was more stable for a gravity bouncing ball than for ATs in a sensorimotor timing task.

The present task was a classic beat synchronization task in sensorimotor timing research (Repp, 2005; Patel, 2014) and the present moving visual stimuli had strong ecological relevance (Gan et al., 2015; Huang et al., 2018; Gu et al., 2019). The current results supported the hypothesized enhancement of visuomotor timing under optimized conditions (Gan et al., 2015). The behavioral auditory over visual superiority is an essential basis for the auditory-specific neural (Thaut et al., 1999; Zatorre et al., 2007; Patel, 2014) and evolutionary (Patel et al., 2009; Patel, 2014) hypotheses of sensorimotor timing. Given the recent observation of improved visuomotor timing performance by moving visual stimuli, reconsideration of the auditory-specific biological substrates of sensorimotor timing has been suggested (Gan et al., 2015; Iversen and Balasubramaniam, 2016). As have been discussed in the Introduction, improved visuomotor timing performance does not necessarily require a renewal of the auditory-specific mechanisms, considering that the brain may utilize the audiomotor pathway to process visual temporal information. The visual over auditory superiority established in the present results, however, would suggest the existence of tight visual-motor cortical coupling in sensorimotor timing.

In addition to the current gravity bouncing ball that was initially devised in Gan et al. (2015), studies of sensorimotor timing have also used other types of moving visual stimuli, such as the moving stimuli with a constant velocity (Hove et al., 2010, 2013a; Varlet et al., 2014), the continuously color changing stimuli (Hove et al., 2010; Varlet et al., 2012), the moving stimuli with human-like velocity profiles (Varlet et al., 2014; Su, 2016), the bouncing ball with a velocity varied according to a rectified sinusoid (Hove et al., 2013b; Iversen et al., 2015), the bouncing ball squashing at the bouncing point (Silva and Castro, 2016), the contracting ring with a uniformly varying velocity (Huang et al., 2018), as well as the gravity bouncing ball with a discontinuous trajectory (Gu et al., 2019). Given the various types of moving visual stimuli described above, several considerations would be further discussed. First, the effect of synchronization improvement is dependent on the types of moving visual stimuli, as well as on the compatibility between motion directions of the moving stimulus and the tapping finger (Hove et al., 2010; Gan et al., 2015). While multiple factors could be associated with the ecological relevance of periodically moving visual stimuli (Huang et al., 2018), the sense of collision at times of beats is common and is considered as a major factor determining the visuomotor improvement (Hove et al., 2013a; Gan et al., 2015; Gu et al., 2019). The gravity bouncing ball that provides a strong sense of collision is so far the most effective stimulus type in improving synchronization to a visual beat. Second, it is worth mentioning that, different from visual timing in which continuous moving visual stimuli outperform discrete static stimuli, the opposite occurs in auditory timing in which discrete static stimuli outperform continuous varying stimuli such as sirens (Hove et al., 2013a).

If there exists a general modality difference, it would indicate that visuomotor timing is unlikely to be substantially improved even the factors other than modality difference also have contributions. On the other side, however, if a general modality difference does not exist, modality difference would be just one factor (though an important one) influencing sensorimotor timing. As have been described, many factors could contribute to sensorimotor timing and we have introduced an ecological view that accounts for ecological relevance of the possible factors (Repp and Penel, 2004). In daily life, people require flexible and effective sensorimotor timing, either in an auditory or in a visual environment. The ecological view suggests ecological relevance of continuously varying visual stimuli and discrete auditory stimuli, which is supported by existing experimental evidence. The specific purpose of the present study was to test the hypothesized visual over auditory superiority in sensorimotor timing. For a fair comparison with respect to the research aim, we adopted the most effective visual stimulus type – the gravity bouncing ball and the most effective auditory stimulus type – the discrete tones. It is possible that, the most effective auditory or visual stimulus type at this time could be further optimized, which would provide further evidence in agreement with the ecological account that sensorimotor timing depends on multiple ecological factors in both auditory and visual environments. For instance, a recent perceptual timing study found that ecological auditory stimuli (i.e., feet tap sound for feet tap video) facilitated perception of a timing deviation in tap dance sequences (Murgia et al., 2017). Consistently, for sensorimotor timing studies employing VB stimuli, it has been recommended to test the natural sound of ball collision (Huang et al., 2018). Therefore, future research is required to further investigate sensorimotor timing processing of ecological sounds, and clarify how the sensorimotor performance is influenced by ecological factors in different natural situations.

In summary, the visual over auditory superiority in a beat synchronization task established in the present study challenges the general auditory superiority in temporal processing and indicates tight visual-motor cortical coupling in sensorimotor timing. The finding suggests that compared to the auditory system, the visual system is also able to work with the motor system to efficiently process temporal information, at least under optimized conditions. Further investigations of the efficiency of visual temporal processing are also required for perceptual tasks and complex temporal patterns, with a focus on the underlying neural substrates (Repp and Su, 2013; Iversen and Balasubramaniam, 2016).

## Data availability statement

The data generated during and/or analyzed during the current study are available from the corresponding author on reasonable request.

## Ethics statement

The studies involving human participants were reviewed and approved by the Institutional Review Board of Psychology Department of Sun Yat-sen University. The patients/participants provided their written informed consent to participate in this study.

## Author contributions

LZ and YH: methodology, software, investigation, formal analysis, and writing – original draft and review and editing. ZG: methodology, software, investigation, and formal analysis. LG and JY: software and investigation. SZ: investigation. XW: conceptualization, methodology, software, formal analysis, writing – original draft review and editing, supervision, and funding acquisition. All authors contributed to the article and approved the submitted version.
